# Ethionine Suppresses Mitochondria Autophagy and Induces Apoptosis via Activation of Reactive Oxygen Species in Neural Tube Defects

**DOI:** 10.3389/fneur.2020.00242

**Published:** 2020-04-07

**Authors:** Li Zhang, Yanting Dong, Wenzhuo Wang, Taoran Zhao, Tingjuan Huang, Ajab Khan, Lei Wang, Zhizhen Liu, Jun Xie, Bo Niu

**Affiliations:** ^1^Department of Biochemistry and Molecular Biology, Shanxi Medical University, Taiyuan, China; ^2^Department of Respiratory and Critical Care Medicine, Second Hospital of Shanxi Medical University, Taiyuan, China; ^3^Beijing Municipal Key Laboratory of Child Development and Nutriomics, Capital Institute of Pediatrics, Beijing, China

**Keywords:** ethionine, neural tube defects, reactive oxygen species, mitochondrial membrane potential, mitochondria autophagy, apoptosis

## Abstract

Abnormal development of central nervous system (CNS) caused by neural tube defects (NTDs) is not only remained the major contributor in the prevalence of stillbirths and neonatal deaths, but also represents a significant cause of lifelong physical disability in the surviving infants. Ethionine is a non-proteinogenic amino acid and antagonist of methionine. Methionine cycle is essential for the elimination of reactive oxygen species (ROS), while lysosomes are involved in the initiation of autophagy. However, its role in ethionine-induced cell death in neural tube defects, still need to be explored. In this study, we investigated the effect of ethionine on NTDs as well as the underlying mechanism involved in this process. Following the establishment of NTDs model using ethionine-induced C57BL/6 mice, ethionine was intraperitoneally injected at a dose of 500 mg/kg in E7.5. Our study revealed that ethionine has induced mitochondrial apoptosis in NTDs by reducing mitochondrial autophagy both *in vivo* and *in vitro*. These results provided a possible molecular mechanism for redox regulation of autophagic process.

## Introduction

The death and disabilities followed by the central nervous system (CNS) diseases are always a major public health concern (1). Neural tube defects (NTDs) are the most common and serious birth defects of CNS resulted due to unclosed or partially closed neural tube. Its phenotypes mainly include anencephaly, spina bifida and encephalocele. The occurrence of neural tube defects is 0.5–2/1,000 in the world, especially in Shanxi province of China it can reach up to 13.9/1,000 (2). The pathogenesis of NTDs is very complex, involving both genetic and environmental factors. Lots of results showed that folic acid deficiency contribute significant risk to NTDs development (3). In recently years, folic acid has emerged as a new therapeutic substance against NTDs (4), however, it cannot prevent all types of NTDs. Therefore, it is urgent to develop a new effective intervention to prevent NTDs. Systemic study is the first step to uncover the potential therapeutic targets for NTDs induced by folic acid deficiency and its specific mechanism of action needs to be investigated.

In the early 1950's, ethionine was first used to study the mechanism of protein biosynthesis. Ethionine is a natural compound which is an S-ethyl analog of methionine. This substitution significantly increased the size and length of the molecule. Due to reduction of SAM during DNAs, RNAs and protein synthesis, it completely changes the action of methionine (5, 6). Recent findings have shown that S-adenosylmethionine (SAM) is a versatile metabolite that promotes cell survival. SAM is synthesized in the cytosol and nuclei but a significant bulk amount (about 30%) is found in the mitochondria (mSAM) where it can play a critical role in the methylation of mitochondrial components (7, 8). Ethionine can also cause NTDs through suppressing the methionine cycle in the whole embryo culture (6). Interestingly, SAM consistently enhances the level of autophagy markers, beclin-1 and LC3B-II (9).

Autophagy is a catabolic process of various organelles, performed to remove unwanted cellular components through double-membrane auto-phagosomes fused to lysosomes (phagolysosome) (10). Studies have shown that autophagy is an essential process for the survival, development and homeostasis of organism (11, 12). Mitochondria are essential for cellular metabolism to synthesize energy in the form of ATPs by oxidative phosphorylation, known as mitochondrial bioenergetics (13). Thus, play a role in cell maintenance and survival, including calcium signaling and storage, metabolite synthesis and apoptosis (14). PINK1-Parkin-mediated mitochondrial autophagy is the most widely studied pathway of mitochondrial autophagy. Studies have shown that as MMP decreases, mitochondrial-specific protease presenilin-associated diamond-shaped protein (PARL) and mitochondrial processing peptidase (MPP) are inactivated, and thus resulted in regulation of PINK1 levels (15). Recent studies have reported that TOMM20 plays an essential role as a receptor for proteins which can target mitochondria (16). However, the mechanism of maternal methionine cycle disorders that represses autophagy during neural tube development is still not clear.

ROS are generated during oxygen metabolism as a byproduct of cellular respiration (17) which can activate intracellular signaling transduction pathways in many CNS diseases, such as inflammation, apoptosis and autophagy (18). Furthermore, mitochondria are both the site of ROS generation in the cell and is also an organelle that is very sensitive to ROS damage (19). It is well-known that ROS formation is essential for autophagy (20). Methionine accumulation increases ROS production in liver and kidney mitochondria (21). Some results also showed that methionine and methionine metabolites such as homocysteine directly modify the rate of mitochondrial ROS production (21). Recently, enormous studies have reported that S-adenosylhomocysteine is highly associated with apoptosis triggered by enhancing ROS and activating caspase-3 (22). However, the reasons for these biological phenomena are poorly understood. We investigated the role of ROS and concluded that methionine inhibitor i.e., ethionine, inhibited autophagy, increased cell apoptosis, and maintained cell survival by increasing ROS in HT-22 cells.

Our hypothesis was that methionine cycle inhibitor-ethionine, may act via modifying gene expression, over-production of reactive oxygen species, reducing mitochondrial membrane potential, enhancing lysosomal alkalization, suppressing mitochondria autophagy, further inducing cell apoptosis and thus participate in the development of NTDs. Based on this assumption, our results revealed that ethionine induced formation of neural tube defects by increasing apoptosis and suppressing autophagy due to reactive oxygen species formation, increasing lysosomal alkalization and reducing mitochondrial membrane potential. We propose that methionine restriction may be a possible intervention for preventing ethionine induced NTDs.

## Materials and Methods

### Animals

C57BL/6 mice weighting 19–26 g from 9 to 11 weeks were obtained from the Animal Laboratory Center of Shanxi Medical University, Taiyuan, People's Republic of China. The procedure was in accordance with the “Guidelines for the Use of Nursing Animals” issued by the National Institutes of Health (8th edition, 2011, revised edition). The experimental protocol was approved by the Experimental Animal Management Committee of Shanxi Medical University. On day 7.5 of pregnancy (E7.5), ethionine (Sigma-Aldrich, USA) was intraperitoneally injected only once at a dose of 0–700 mg/kg to establish the NTDs embryo model in C57BL/6 mouse. The same dose of sesame 0.1 mmol/l NaOH was intraperitoneally injected to the pregnant mice for control group.

### Cell Culture and Treatments

HT-22 cells were grown in DMEM (Hyclone, Logan, UT, USA) supplemented with 10% fetal bovine serum (FBS, Gibco, USA). Cells were incubated in a humidified atmosphere at 37°C with 5% CO_2_, treated with 20 mmol/L ethionine (Sigma-Aldrich, USA) and 10 μmol/l rapamycin (MCE, USA), respectively.

### TUNEL Staining Assay

Apoptosis was assessed using an *in situ* Cell Death Detection kit, POD (Roche, 11684817910, USA). A 5 μm embryonic tissues were sectioned, paraffin was removed, incubated and cells were permeated at 37°C for 20 min. The sections were washed with PBS thrice, 3% hydrogen peroxide was used for 20 min to stop endogenous peroxidase activity, and washed again three times with PBS. The slides were dried and 50 μl TUNEL reaction mixture (5 μl TDT + 45 μl fluorescein-labeled dUTP solution) was added to each section and incubated at 37°C for 40 min. Post washing with PBS (three times), 50 μl POD was added to each section and was incubated again for 30 min. Again five times washing with PBS, 50 μl DAPI was added to each section and incubated at room temperature for 8 min and were studied using a light microscope (Nikon, Tokyo, Japan). The ImageJ software (Plugins-Analyze-Cell counter) was used, and the green fluorescence cells which are considered as TUNEL positive cells were counted in three different microscopic fields.

### Quantitative RT-PCR (qRT-PCR)

Total RNA was extracted from brain tissues using TRIzol reagent (Invitrogen, Carlsbad) and cDNA was synthesized using Revert Aid First Strand cDNA Synthesis Kit (Thermo, USA). Quantitative PCR was performed on a Real-Time PCR platform (Applied Biosystems, USA) in 20 μl reaction mixture containing 10 μl 2 × SYBRPremix Ex Taq (Takara, Japan), 0.5 μl primers (2.5 μM), 2 μl cDNA, and 7 μl ddH2O. The data was analyzed with 2^−ΔΔCt^ method and the mRNA level was normalized with β-actin. The sequences of primers used for mouse cDNA were as follows: P62, 5′-AGGAGGAGACGATGACTGGACAC-3′ and 5′-TTGGTCTGTAGGAGCCTGGTGAG-3′, Beclin-1, 5′- ATGCTGTCCTCCCGTTCCTCTG−3′ and 5′- CCTGGTCCTGCTGCGTTGATG-3′, ATG5, 5′-AGTCAAGTTCAGTGGAGGCAACAG-3′ and 5′- GTGTCTCAGCGAAGCAGTGGTG-3′, ULK1, 5′-CGGACCAGGCAGACATTGAGAAC-3′ and 5′-AGGTTGGCAGCAGGTAGTCAGG-3′, β-actin (B661302-0001, sangon, Shanghai).

### Determination of ROS Generation

A change in intracellular ROS generation was assessed using DCFH-DA (Beyotime, Shanghai, China) by flow cytometry. Cells were incubated with ethionine or MnTMPyP (Sigma-Aldrich, USA). The cells were collected, washed with PBS and incubated with DCFH-DA for 25 min at 37°C. Then DCFH fluorescence distribution of 10,000 cells was detected by flow cytometry (BD Biosciences, San Jose, CA, USA). In addition, HT-22 cells (1 × 10^4^ cells/well) were seeded in 24-well plate and post 48 h of incubation with ethionine or MnTMPyP, cells were visualized with a fluorescence microscope (Nikon, Tokyo, Japan).

### Measurement of Mitochondrial Membrane Potential

Experiments were conducted by JC-1 fluorescence (Beyotime, Shanghai, China) using flow cytometry. HT-22 cells were collected in a 15 ml microcentrifuge tube, and were resuspended in 0.5 ml DMEM and 5% FBS with JC-1 dye working solution. Cells were incubated in 37°C and 5% CO_2_ for 20 min, washed twice with PBS and cellular fluorescence was detected by flow cytometer. In addition, 1 × 10^4^ cells/well were seeded in 24-well plates and after 48 h of incubation with ethionine, rapamycin or MnTMPyP, cells were washed twice with 1 × JC-1 buffer and incubated again with JC-I dye working solution at 37°C for 20 min. After washing twice with 1 × JC-1 buffer, the cells were visualized with a fluorescence microscope (Nikon, Tokyo, Japan).

### Acridine Orange Staining

Acridine Orange (AO) (Solarbio, Beijing, China) is an important weak alkaline stain for detecting the structure of acidic vesicles. Cells in 6-well culture dishes were incubated with ethionine and MnTMPyP. Then, cells were washed twice with PBS, and then resuspended in 0.5 ml DMEM and 5% FBS with acridine orange dye working solution for 15 min at 37°C. The cells were visualized using fluorescence microscope (Nikon, Tokyo, Japan) and the data were analyzed with ImageJ Software.

### Cell Apoptosis Assay

Annexin V-FITC/PI apoptosis detection kit (KeyGEN, Suzhou, China) was also used for cell apoptosis assay. Cells were treated with ethionine for 48 h and resuspended in 500 μL binding buffer and dyed with 5 μL Annexin V-FITC and 5 μL PI for 15 min at room temperature, and cell apoptosis in each group was detected by flow cytometry.

### Immunofluorescence Analysis

Cells were seeded in 24-well plate, treated with ethionine and rapamycin for 48 h, and cells were identified by standard immunofluorescence staining. The following primary antibodies were used: anti-TOMM20 (1:100; ABclonal, A19403), and secondary antibodies used as Goat Anti-rabbit IgG H&L (Invitrogen, A11011). Nuclei were counterstained with DAPI (Sigma-Aldrich). Images were captured using a fluorescence microscope (Nikon, Tokyo, Japan).

### Western Blotting Analysis

The cells or embryo brain tissues were lysed with RIPA lysis buffer (Solarbio, Beijing, China) supplemented with a protease inhibitor cocktail (Sigma-Aldrich, USA) and 10 mM PMSF (Solarbio, Beijing, China). 20 μg proteins were electrophoresed on 12% SDS-PAGE and electro-transferred into PVDF membranes (Millipore, Billerica, MA, USA). After blocking with 5% skim milk in PBST (PBS with 0.05% Tween-20) for 1 h at room temperature, the membranes were incubated with primary antibodies overnight at 4°C. Followed incubation with secondary antibody at room temperature for 2 h, bands were visualized using an enhanced chemiluminescent (ECL) blot detection system (ChemiDoc^TM^ Imaging Systems, BIO-RAD, USA) following the manufacture's instruction. The protein bands were quantified using ImageJ software, and β-Tublin was used as a housekeeping control. The primary antibodies used were mouse anti-LC3B (1:1000; ab63817, Abcam, Massachusetts, US), rabbit anti-Beclin-1 (1:1500; ab62557, Abcam, Massachusetts, US), mouse anti-P62 (1:1000; ab56416, Abcam, Massachusetts, US), rabbit anti-PINK1 (1:2000; ab23707, Abcam, Massachusetts, US), rabbit anti-Parkin (1:1000; AF0235, Affinity, China), rabbit anti-Cleaved Caspase-3 (1:1000; #9664, Cell Signaling Technology, US), rabbit anti-CTSB (1:1000; ab214428, Abcam, Massachusetts, US), rabbit anti-BAX (1:2000; ab32503, Abcam, Massachusetts, US), rabbit anti-BCL-2 (1:1000; ab196495, Abcam, Massachusetts, US), rabbit anti-TOMM20 (1:1000; A19403, ABclonal, China), rabbit anti-β-Tublin (1:1000; Abcam, Massachusetts, US) and secondary antibodies were goat anti–rabbit IgG (1:3000; ZB-2301; ZSGB-BIO, Beijing, China), and goat anti–mouse IgG (1:3000; ZB-2301; ZSGB-BIO, Beijing, China).

### Statistical Analysis

SPSS17.0 was used for data analysis. Statistical differences were performed by Student's *t*-test for two group comparisons and one-way ANOVA for more than two group comparisons. Moreover, in one-way ANOVA analyses, LSD *t*-test were used to estimate the significance of the results. Differences were considered statistically significant when the ^*^p < 0.05, ^**^p < 0.01, or ^***^p < 0.001.

## Results

### Ethionine Induces Neural Tube Defects

**Figure 1:**
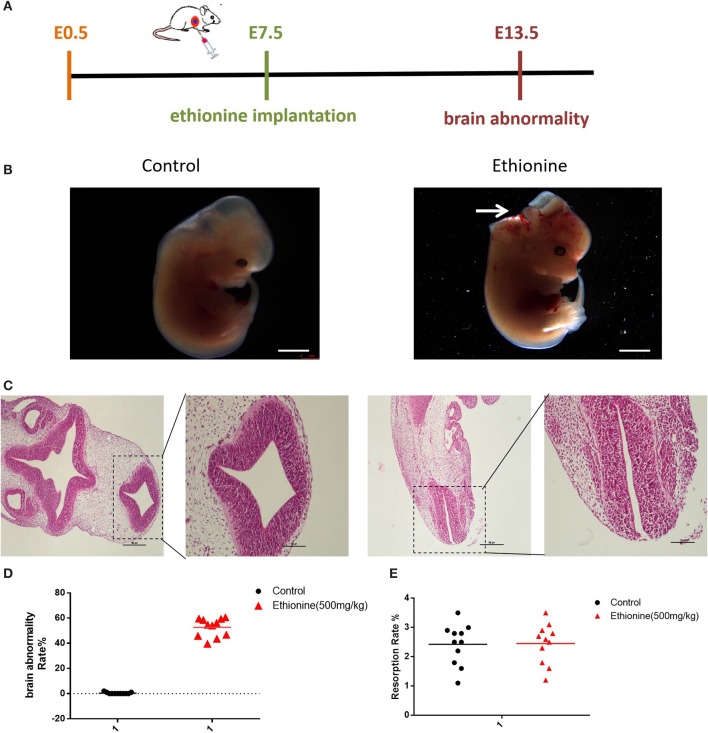
Ethionine induced NTDs. **(A)** The ethionine treatment schematic diagram. Ethionine was injected intraperitoneally at E7.5 and embryos were isolated and extracted at E13.5. **(B)** Morphology of E13.5 embryos and histological view of the neural tubes (NT) 50 ×. **(C)** Photomicrographs of coronal sections of normal embryos and NTD embryos using hematoxylin and eosin staining. Scale bars: 100 μm. **(D)** Brain abnormality rates in C57BL/6 mice with or without ethionine. Incidence rates were calculated by embryos. **(E)** Absorption rate in C57BL/6 mice with or without ethionine. Each experiment was carried out in triplicate (*n* = 11).

All embryos were isolated and observed under a stereo microscope on E13.5 (Figure 1A). The results showed that with increased dose of ethionine, the rate of embryonic resorption and growth retardation was also increased. At 700 mg/kg ethionine, all embryos were resorbed, while at 500 mg/kg, the incidence of NTDs was at highest rate (54.8%) with a lower embryonic resorption rate (8.2%). Based on these results, we selected 500 mg/kg as an optimal dose to establish NTDs model in the present study (Table 1). The structural characteristics of tissues in the control embryos showed full appearance while that of NTDs embryos showed abnormal brain development (Figure 1B). In NTDs embryo, serial histological sections revealed that neural tube closure was failed in the hindbrain region (Figure 1C). These findings established a pivotal role for ethionine in NTDs and highlighted the importance of appropriate level of methionine in NTDs. The NTDs rate (56.42 ± 2.24%) in embryos with ethionine was significantly increased compared with control group (0.3636 ± 0.2033%) (Figure 1D). However, no difference was observed in the resorption rates between control and ethionine treatment groups (Figure 1E).

**Table 1:** Embryonic phenotypes of mice treated with ethionine.

**Ethionine** **(mg/kg)**	**Pregnant mice** **(*n*)**	**Embryos** **(*n*)**	**Normal** ***n* (%)**	**Resorption** ***n* (%)**	**Growth retardation** ***n* (%)**	**NTDs** ***n* (%)**	**Other malformation** ***n* (%)**
0	14	89	82 (92.2)	5 (5.6)	2 (2.2)	0 (0)	0 (0)
50	7	41	36 (87.8)	4 (9.8)	1 (2.4)	0 (0)	0 (0)
100	6	35	28 (80.0)	4 (11.4)	3 (8.6)	0 (0)	0 (0)
200	10	69	39 (56.6)	13 (18.8)	9 (13)	8 (11.6)	0 (0)
400	9	56	29 (51.8)	0 (0)	17 (30.3)	8 (14.3)	1^a^,1^b^ (3.6)
500	11	73	6 (8.2)	6 (8.2)	18 (24.7)	40 (54.8)	2^a^,1^b^ (4.1)
600	7	45	2 (4.4)	38 (84.5)	2 (4.4)	3 (6.7)	0 (0)
700	9	53	0 (0)	100 (100)	N/A	N/A	N/A

a *Craniofacial malformation*.

b *Polydactyly*.

### Ethionine Increases Apoptosis and Suppresses Autophagy

**Figure 2:**
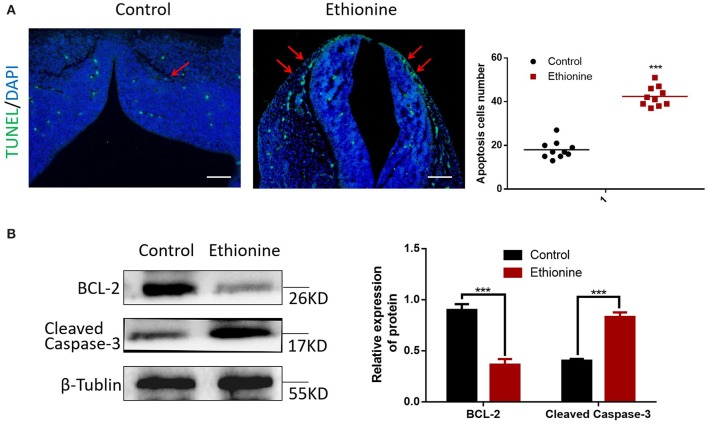
Ethionine induced cell apoptosis in normal and NTD embryos. **(A)** Representative TUNEL assay images showing apoptotic cells (Green dots) in E13.5 embryos and quantification of TUNEL positive cells (*n* = 3, ***P < 0.001 vs. control group). Cell nuclei were stained with DAPI (blue). Scale bars: 500 μm. Data were analyzed with ImageJ Software. **(B)** BCL-2 and Cleaved Caspase-3 protein levels in control and ethionine groups were evaluated via Western blotting. β-Tublin levels were also evaluated to confirm equal loading (*n* = 3). Each experiment was carried out in triplicates ***P < 0.001 vs. control group.

**Figure 3:**
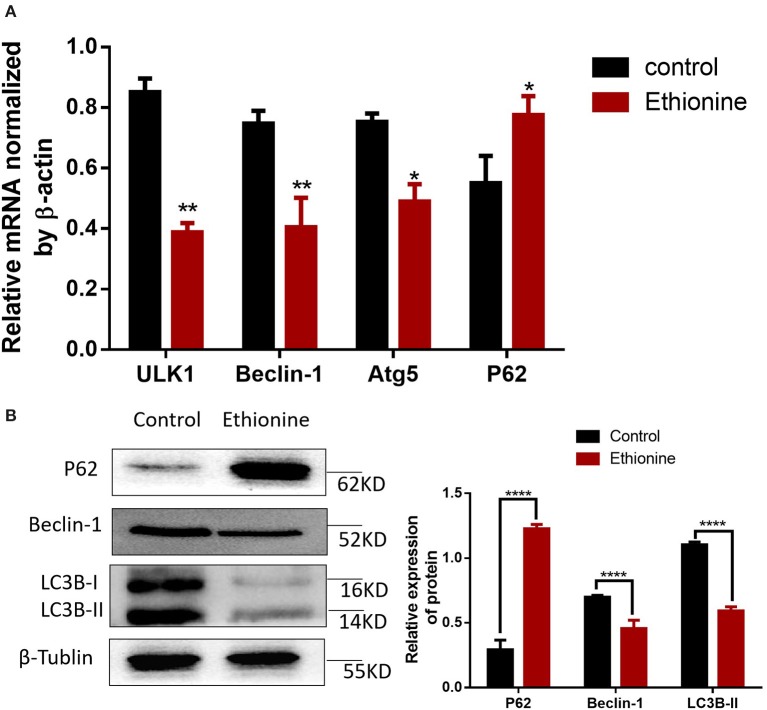
Ethionine suppressed autophagy in normal and NTD embryos. **(A)** mRNA level of autophagy related genes were analyzed by qRT-PCR in normal and NTD embryos. The β-actin gene was used as a control. **(B)** P62, Beclin-1 and LC3B protein levels in control and ethionine groups were evaluated via Western blotting. β-Tublin levels were also evaluated to confirm equal loading (*n* = 5), Each experiment was carried out in triplicates *P < 0.05, **P < 0.01, and ****P < 0.0001 vs. control group.

To calculate the level of apoptosis in ethionine-induced embryos, we investigated the ratio of TUNEL-positive cells on serial histological sections. There was an obvious increase in TUNEL-positive cell numbers in ethionine-induced embryos compared with normal one. Ethionine induced excessive apoptosis in the embryos treated with ethionine (Figure 2A). Furthermore, we evaluated that there was a significant up-regulation and down-regulation of Cleaved Caspase-3 and BCL-2 protein, respectively in ethionine-induced embryos compared with the control group (Figure 2B). Firstly, ethionine suppressed the expression of ULK1, Atg5, and Beclin1 as shown in Figure 3A which are essential for autophagosome formation. Simultaneously, ethionine also increased the expression of p62 (Figure 3A) which negatively regulates the process of autophagy and is an indicator of impaired autophagy. Thus, ethionine induced autophagy by altering gene expression that regulates autophagy. Moreover, there was an obvious decreased in the level of LC3B-II (microtubule-associated protein 1 light chain 3) and Beclin-1 in ethionine-induced embryos (Figure 3B). In contrast, the level of P62 was increased in ethionine-treated group compared with the control group (Figure 3B), suggesting that ethionine has inhibited autophagy, causes excessive apoptosis and thus resulted in programmed cell death.

### Ethionine Induces Reactive Oxygen Species

**Figure 4:**
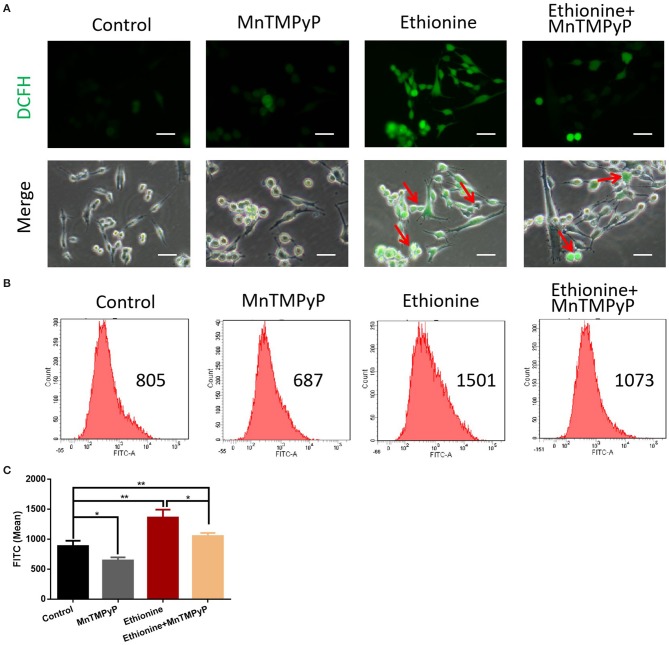
Ethionine induced reactive oxygen species. **(A)** HT-22 cells were treated with ethionine and MnTMPyP, and ROS levels were determined using DCFH-DA. Representative images are shown. Scale bars: 100 μm. **(B)** Changes in intracellular ROS generation was assessed using DCFH-DA by flow cytometry. **(C)** Quantification of ROS analysis. One-way ANOVA followed by LSD *t*-test were used for difference compared with other groups. *P < 0.05 and **P < 0.01.

To test whether ROS play a role in autophagy, we measured ROS production under ethionine treatment using a fluorescent probe, DCFH-DA. The toxic effect of ethionine was showed to be mediated by ROS overproduction (Figure 4A). We next evaluated the expression of ROS, its role in autophagy, and to investigate the mechanism by which ethionine exerts its neurological effect. Compared with control conditions, ethionine treatment significantly increased ROS levels (Figures 4B,C). Interestingly, the level of ROS was reduced when cells were incubated with both ethionine and oxidative stress inhibitor-MnTMPyP. Thus, ethionine has increased oxidative stress leading to mitochondrial dysfunction and the effect was improved when ethionine and MnTMPyP were present.

### Ethionine Induces Lysosomal Alkalization

**Figure 5:**
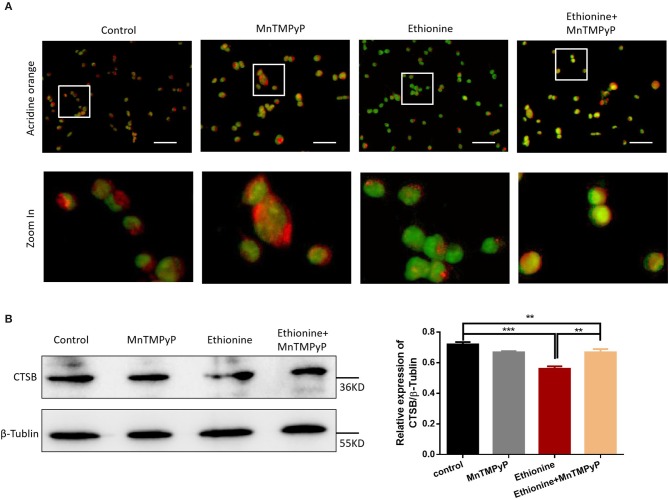
Ethionine induced lysosomal alkalization. **(A)** HT-22 cells were treated with ethionine and MnTMPyP, and lysosomal alkalization levels were determined using AO dye. Representative images are shown. Scale bars: 100 μm. **(B)** CTSB protein level in control and experiment groups was evaluated via Western blotting. β-Tublin levels were also evaluated to confirm equal loading (*n* = 3), Each experiment was carried out in triplicates **P < 0.01 and ***P < 0.001.

To elucidate the mechanism of ethionine which block cellular and mitochondrial autophagy, AO fluorescence was used. As shown in Figure 5A, ethionine significantly increased the proportion of green fluorescent vesicles, suggesting that ethionine promoted alkaliization of HT-22 cells vesicles. Simultaneously, we also found that MnTMPyP improved this phenomenon and reduced cell lysosomal alkalinity. Furthermore, overproduction of ROS is an important mechanism for ethionine-induced lysosomal alkalization. In addition to this, protease-CTSB is important for autophagy degradation and the results showed that ethionine treatment significantly decreased CTSB protein level compared with the control group (Figure 5B). These results suggested that ethionine inhibited lysosomal function by affecting the level of CTSB in lysosomes. Similar to the process of ethionine-induced lysosomal alkalization, there was an obvious increased in the ethionine treated group compared with control group.

### Ethionine Reduces MMP by Increased Production of ROS

**Figure 6:**
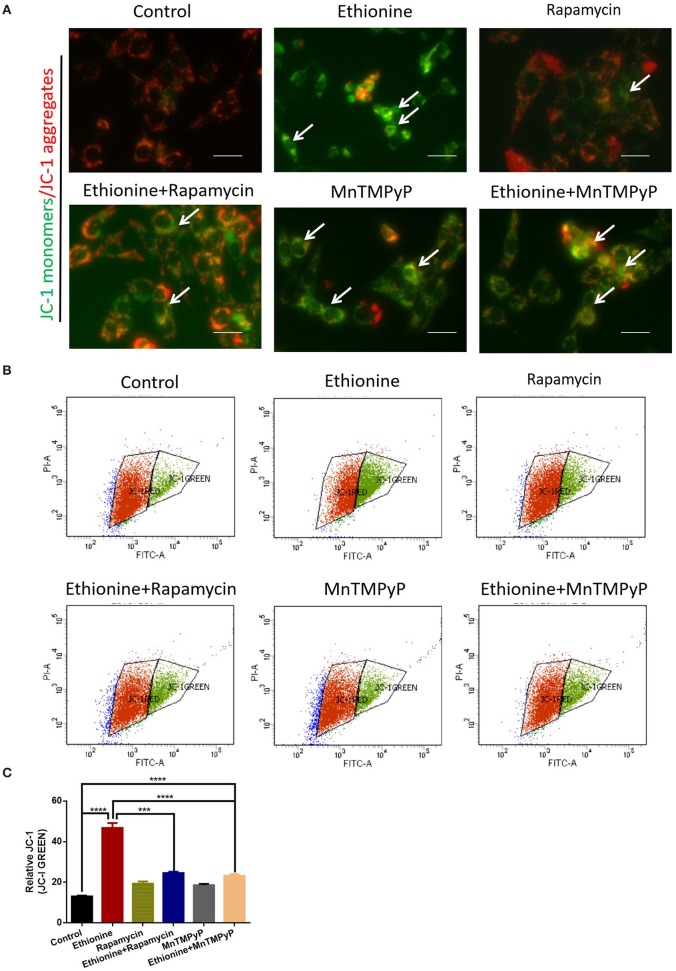
Ethionine suppressed mitochondrial membrane potential. **(A)** HT-22 cells were treated with ethionine, rapamycin and MnTMPyP, and MMP level was determined using JC-1 dye. Monomers were stained with green and aggregates were stained with red. Representative images are shown. Scale bars: 500 μm. **(B)** MMP levels were assessed using JC-1 fluorescence by flow cytometry. **(C)** Quantification of MMP analysis. Each experiment was carried out in triplicates. ***P < 0.001 and ****P < 0.0001.

To determine the pro-apoptotic mechanism of ethionine on brain abnormality, mitochondrial function and ROS generation was monitored. MMP showed that cells in the control group exhibited high mitochondrial potential as evident from more red fluorescence while ethionine treatment has reduced MMP by exhibiting more green fluorescence after the administration of JC-1 probe as shown in Figure 6A. For further confirmation, the MMP was also analyzed by flow cytometry and the data from JC-1 assay illustrated that ethionine has increased the ratio of green/monomeric forms of JC-1 in HT-22 cells (Figures 6B,C). These results suggested that ethionine has successfully blocked mitochondrial autophagy.

### Ethionine Inhibits PINK1-Parkin-Mediated Mitochondrial Autophagy

**Figure 7:**
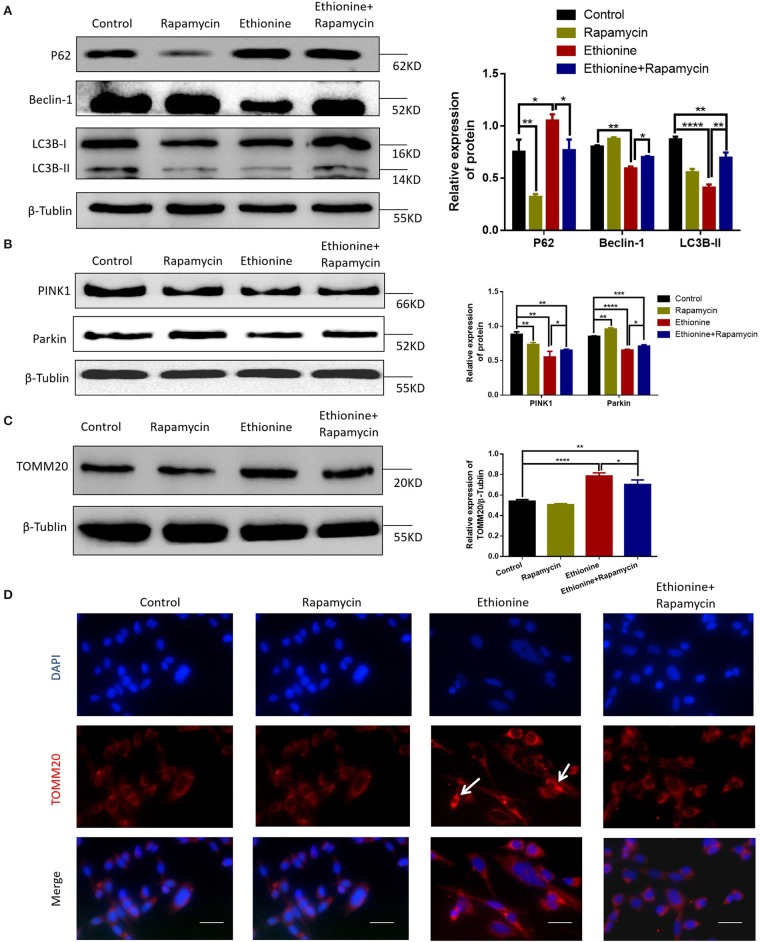
Ethionine inhibited PINK1-Parkin-mediated mitochondrial autophagy. **(A)** HT-22 cells were treated with ethionine and rapamycin, autophagy related mark-LC3B, P62, Beclin-1 protein level in control and experiment groups were evaluated via Western blotting. **(B)** PINK1, Parkin protein levels in control and experiment groups were evaluated via Western blotting. **(C)** TOMM20 protein levels in control and experiment groups were evaluated via Western blotting. For all β-Tublin level was evaluated to confirm equal loading (*n* = 3). One-way ANOVA followed by LSD *t*-test were used for difference compared with other groups, *P < 0.05, **P < 0.01, ***P < 0.001, and ****P < 0.0001. **(D)** Fluorescence microscopy analysis of TOMM20 (red) staining in HT-22 cells after treatment with ethionine and rapamycin for 48 h. Nuclei were also stained with DAPI, and representative single optical sections and merge images are shown. Scale bars: 500 μm. Each experiment was carried out in triplicates.

To investigate the role of ethionine in impairing mitophagy, we assessed the levels of autophagy related proteins and competence of PINK1-Parkin-mediated mitochondrial autophagy. LC3B-II and Beclin-1 levels were reduced in ethionine treatment group compared with control group (Figure 7A), suggesting impaired mitophagy. In addition to this, we investigated the effect of ethionine on PINK1-Parkin-mediated mitochondrial autophagy, which showed that mitochondrial proteins including PINK1 and Parkin were downregulated in ethionine treatment group compared with control one (Figure 7B). To further confirm that ethionine has a blocking effect on mitochondrial autophagy degradation, the mitochondrial proteins TOMM20 (translocase of outer mitochondrial membrane 20) were not degraded with this treatment (Figure 7C) and was obviously increased in ethionine treatment group (Figure 7D). Results showed that ethionine inhibited autophagy degradation process through reducing PINK1-Parkin-mediated mitochondrial autophagy.

### Ethionine Activate Caspase-3-Dependent Mitochondrial Apoptosis

**Figure 8:**
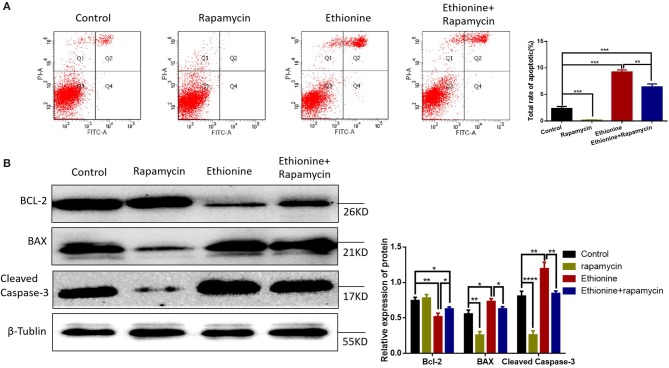
Ethionine activated Caspase-3-dependent mitochondrial apoptosis. **(A)** HT-22 cells were treated with ethionine and rapamycin, the cell apoptosis was analyzed by flow cytometry. **(B)** BAX, BCL-2, and Cleaved Caspase-3 protein levels in control and experiment groups were evaluated via Western blotting. β-Tublin levels were also evaluated to confirm equal loading (*n* = 3). Each experiment was carried out in triplicates, *P < 0.05, **P < 0.01, ***P < 0.001, and ****P < 0.0001.

To understand the significance of ethionine during neurogenesis, we investigated the effect of ethionine on cell apoptosis in HT-22 cells. Ethionine treatment group showed high rate of early and late apoptosis compared with the control group (Figure 8A) and was reduced in rapamycin treatment group. Flow cytometry indicated that ethionine significantly promoted while rapamycin suppressed cell apoptosis. A significant up-regulation of BAX and Cleaved Caspase-3 and down-regulation of BCL-2 proteins were detected after ethionine treatment as shown in Figure 8B.

## Discussion

NTDs are the most common global birth defects. Human clinical trials and population studies have shown that folate contents are associated with risk for NTDs (23, 24). However, there is no effective pharmacological target, as well as specific pathogenesis is not clear for brain abnormalities. The overall objective of the current study was to understand the complicated effects of ethionine on neural tube closure which might be the foundation for resolving the pathogenesis of NTDs. Our study has confirmed that mitochondria are important targets for ethionine neurotoxicity in ethionine-induced embryos. Based on this, we further evaluated the potential mechanism of ethionine on mitochondrial toxicity in HT-22 cells. We evaluated that ethionine has suppressed autophagy by increasing the level of P62, and reducing the level of LC3B and Beclin-1 in ethionine-induced embryos and HT-22 cells while significantly reduced the expression of CTSB. Simultaneously, ethionine has significantly increased the expression of mitochondrial marker TOMM20 and induced Caspase-3-dependent apoptosis. Overall, our findings suggested that methionine cycle inhibitor-ethionine has increased ROS formation, reduced MMP and enhanced lysosomal alkalization, decreased expression of lysosomal cysteine-CTSB which further suppressed mitochondrial autophagy degradation and induced cell apoptosis in neural tube closure.

Firstly, we established a mouse NTD embryo model by injecting ethionine intraperitoneally at E7.5. The results showed that ethonine induced 54.8% NTDs at a dose of 500 mg/kg which is consistent with the Dunlevy et al. (6) and Leung et al. (25). These observations have proved that ethionine has caused failure of the neural tube closure.

Previous studies have shown that methionine is the main target of ROS (26). In our study, we evaluated the expression of ROS by flow cytometry and immunofluorescence to investigate the mechanism of ethionine which showed that ethionine has induced ROS formation. At present, many studies have confirmed that ROS activates autophagy under starvation (20). However, our results confirmed that ethionine has inhibited autophagy through overproduction of ROS. Research has shown that SAM was able to antagonize excessive production of ROS induced by ethanol (27), and ethanol has been reported to inhibit the methionine synthase (28). Our findings also showed that ROS level has been elevated by inhibiting methionine cycle. Furthermore, ethionine has stimulated the excessive production of ROS by causing methionine aggregation and decreasing SAM level, causing abnormal methylation, such as histone, N6-methyladenosine (m6A) RNA and DNA methylation abnormality. Disturbances in single nucleotide, methyl compound and nucleic acid metabolism can cause abnormalities in redox reactions and oxidative phosphorylation, thereby inhibiting ATP production. In addition, we further examined the molecular mechanisms and found that ethionine-induced dysfunction of the mitochondria and lysosomes. Ethionine inhibited the autophagy flow degradation by reducing MMP, increased lysosomal alkalization, and inhibited autophagy. But starvation may induce production of ROS by mitochondrial complex I, causing activation of autophagy (20). To further clarify the specific mechanism, we detected lysosomal alkalization and CTSB expression. Lysosomes are key organelles that decompose and remove excess or damaged cytoplasmic substances from autophagosomes. The present study supports that many autophagy disruptions are related to lysosomal dysfunction (29). AO fluorescence results showed that ethionine promoted lysosomal alkalization in HT-22 cells. Western blotting also confirmed overexpression of ROS with downregulation of CTSB expression caused by ethionine. Over-produced ROS prevents cells from effectively clearing damaged mitochondria in neurons, causing neuronal death (30). Therefore, we further investigated the effect of ROS on lysosomal function of ethionine using osmotic antioxidant MnTMPyP. MnTMPyP treatment significantly inhibited ethionine-induced decrease of CTSB expression. These results prove that ROS overproduction is a necessary mechanism for ethionine-induce lysosomal alkalization, and lysosomes acts as a target of ethionine to interfere with mitochondrial autophagy.

Several studies have reported that autophagy is essential for neurulation or neural tube closure (31). A recent study has reported that mitochondrial autophagy is a specific autophagy process that targets mitochondria and helps to clear damaged, dysfunctional and potentially cytotoxic mitochondria. Research also have showed that methionine is accompanied by activation of autophagy (32). Moreover, the end step of caspase activation is the Cleaved Caspase-3 which is the measure of apoptosis. Previous studies have shown that apoptosis can increase the occurrence of NTDs (33–35). Folic acid deficiency causes inhibition of autophagy, is not completely solved. This study evaluated that ethionine induced apoptosis by performing TUNEL staining on tissue sections, in which more TUNEL-positive cells were observed in ethionine-induced embryos neural tissues than in control neural tissues. At the same time, the expression of autophagy related indicators-LC3B, P62, Beclin-1, and apoptosis-related markers BCL-2 and Cleaved Caspase-3 were also observed. Results showed a significant up-regulation of Cleaved Caspase-3 and P62 while the level of BCL-2, LC3B, and Beclin-1 was reduced in ethionine-induced embryos compared with the normal embryos. Similarly, excessive apoptosis and reduced autophagy were appeared in NTDs embryos. However, western blotting results showed that LC3B-II protein expression level was higher in control group than the rapamycin treatment group in HT-22 cells. We concluded that the treatment concentration of rapamycin was too low, which have no significant effect on the activation of autophagy as the expression level of LC3B-II was not obvious. Results also showed that when ethionine was treated alone, the protein level of LC3B-II was significantly reduced, and when co-treated with ethionine and rapamycin, the protein level of LC3B-II had shown a significantly increase, considering that rapamycin may have a rescue effect on ethionine toxicity. These findings suggest that these events may be causal events in folate-deficient brain abnormality. Subsequent studies were performed to find out the relationship between reduced autophagy/enhanced apoptosis and neural tube closure.

The damaged mitochondria might lead to neural tube abnormal development. The PINK1-Parkin-mediated pathway is one of the classical pathways that regulate mitochondrial autophagy (36). PINK1 and Parkin are Parkinson's disease associated genes, and involved in nervous system development (37). Keeping in view, we detected that the level of PINK1 and Parkin by Western blotting which indicate that the mitochondria were damaged by ethionine, and PINK1 cut down through a loss in mitochondrial potentials. Whether specific genomic loci or others such as proteins or RNA are the specific targets, still needs to be determined.

Our results showed that ethionine suppressed mitochondrial autophagy by reducing MMP and increasing lysosomal alkalization for neural tube closure. Moreover, methionine restriction prolongs lifespan in different species and has beneficial effects on metabolic health and inflammatory response (38). Our data showed that ethionine has suppressed autophagy in ethionine-induced embryos and HT-22 cells. Based on our research data and literature reports, we believed that ethionine inhibited autophagy. On the one hand, ethionine has blocked mitochondrial autophagy flow by inducing excessive production of ROS, reduce MMP, increase mitochondrial lysosome alkalization, decrease expression of lysosomal cysteine-CTSB, and raise the level of mitochondrial proteins TOMM20. On the other hand, ethionine inhibited autophagy degradation through excessive aggregation of methionine, causing direct damage to mitochondria.

## Conclusion

In summary, our study revealed a mechanism underlying ethionine-suppressed mitochondrial autophagy in NTD formation which is focused on ethionine induced mitochondrial apoptosis by reducing mitochondrial autophagy both *in vivo* and *in vitro*, and thus provides a molecular mechanism for redox regulation of autophagic process. However, our current study only explored the potential effect of ethionine on HT-22 cell function, and future studies are needed to validate the effects of autophagy on NTDs in the clinical sample which is important for further investigation of brain developmental pathogenesis.

## Data Availability Statement

All datasets generated for this study are included in the article/supplementary material.

## Ethics Statement

The animal study was reviewed and approved by Animal Laboratory Center of Shanxi Medical University.

## Author Contributions

LZ wrote the manuscript. YD and AK helped in revising the manuscript. TZ analyzed the data. TH, LW, and ZL contributed in figure designing. WW contributed to the manuscript for literature research. JX and BN revised and approved the manuscript. All authors read and approved the manuscript for submission.

### Conflict of Interest

The authors declare that the research was conducted in the absence of any commercial or financial relationships that could be construed as a potential conflict of interest.

## Abbreviations

NTDs: Neural tube defects
ROS: Reactive oxygen species
MMP: Mitochondrial membrane potential
CNS: central nervous system
SAM: S-adenosylmethionine
JC-1, 5, 5′, 6: 6′-Tetrachloro-1, 1′, 3, 3′-tetraethylbenzimi-dazolylcarbocyanineiodide
TOMM20: translocase of outer mitochondrial membrane 20
TUNEL: Terminal Deoxynucleotidyl Transferase dUTP Nick End Labeling
DMEM: Dulbecco's Modified Eagle's Medium
MnTMPyP: Mn (III) tetrakis (1-methyl-4-pyridyl) porphyrin pentachloride
DCFH-DA, 2′: 7′-Dichlorofluorescin diacetate
AO: Acridine orange
PVDF: polyvinylidene fluoride
CTSB: Cathepsin B.
